# Natural variations in expression of regulatory and detoxification related genes under limiting phosphate and arsenate stress in *Arabidopsis thaliana*

**DOI:** 10.3389/fpls.2015.00898

**Published:** 2015-10-23

**Authors:** Tapsi Shukla, Smita Kumar, Ria Khare, Rudra D. Tripathi, Prabodh K. Trivedi

**Affiliations:** ^1^C.S.I.R.-National Botanical Research Institute, Council of Scientific and Industrial ResearchLucknow, India; ^2^Academy of Scientific and Innovative ResearchNew Delhi, India; ^3^Department of Biochemistry, University of LucknowLucknow, India

**Keywords:** *Arabidopsis*, arsenic, gene expression, natural variations, phosphate, transcription factors

## Abstract

Abiotic stress including nutrient deficiency and heavy metal toxicity severely affects plant growth, development, and productivity. Genetic variations within and in between species are one of the important factors in establishing interactions and responses of plants with the environment. In the recent past, natural variations in *Arabidopsis thaliana* have been used to understand plant development and response toward different stresses at genetic level. Phosphorus deficiency negatively affects plant growth and metabolism and modulates expression of the genes involved in Pi homeostasis. Arsenate, As(V), a chemical analog of Pi, is taken up by the plants via phosphate transport system. Studies suggest that during Pi deficiency, enhanced As(V) uptake leads to increased toxicity in plants. Here, the natural variations in *Arabidopsis* have been utilized to study the As(V) stress response under limiting Pi condition. The primary root length was compared to identify differential response of three *Arabidopsis* accessions (Col-0, Sij-1, and Slavi-1) under limiting Pi and As(V) stress. To study the molecular mechanisms responsible for the differential response, comprehensive expression profiling of the genes involved in uptake, detoxification, and regulatory mechanisms was carried out. Analysis suggests genetic variation-dependent regulatory mechanisms may affect differential response of *Arabidopsis* natural variants toward As(V) stress under limiting Pi condition. Therefore, it is hypothesized that detailed analysis of the natural variations under multiple stress conditions might help in the better understanding of the biological processes involved in stress tolerance and adaptation.

## Introduction

Diverse spectrum of environmental stresses severely affects plant growth and development and thus reduces productivity and yield. Several studies have reported that the genetic variations within and in between the species play role in establishing interactions and responses of plants with the environment. In recent years, natural variations in different plant species such as *Arabidopsis*, maize, and rice have been used to understand the genetic impact on plant development and physiology (Alonso-Blanco et al., 2009; Li et al., 2012; Weigel, 2012; Yadav et al., 2013). Apart from the developmental studies, natural variations have also been used to study the effect and response of different accessions under stress conditions (Stein and Waters, 2012). Advanced studies using genetically divergent populations within the species have helped in the elucidation of the genomic variations and their associations with various traits and adaptability. However, among the other plant species *Arabidopsis thaliana* has easily established itself as a tool for the evolutionary and ecological studies due to its number of features (Turck and Coupland, 2013) such as a small genome size and the ease with, which it can be manipulated (Koornneef and Meinke, 2010). In addition, *Arabidopsis* natural variations have been used to elucidate the molecular mechanisms and processes involved in various stresses including salt (Wang et al., 2013), drought (Bouchabke et al., 2008), temperature (Degenkolbe et al., 2012; Barah et al., 2013), and flooding (Vashisht et al., 2011).

Natural and human-induced factors like industrialization; mining, agricultural practices have resulted in the release of detrimental pollutants including toxic heavy metals in the environment. Toxic heavy metals cause drastic changes in the growth, physiology, and metabolism of plants (Finnegan and Chen, 2012). Heavy metals not only hamper plant growth and productivity but also cause severe human health hazards due to the food chain contamination. One such ubiquitous pollutant is arsenic (As), which is widely distributed in the environment. Arsenic occurs in two inorganic forms, arsenite [As(III)] and arsenate [As(V)] of which As(V) can be readily reduced to As(III) after entering into the plant cell. Both these inorganic forms disrupt plant metabolism but through distinct mechanisms (Finnegan and Chen, 2012). As(V) is chemically analogous to inorganic phosphate (Pi) and therefore, is taken up by the plant roots from soil via Pi transport system (Raghothama, 1999; Catarecha et al., 2007; Wu et al., 2011; Castrillo et al., 2013). Inside the plant cell, it replaces PO_4_^-^ from ATP, resulting in the inhibition of ATP synthesis and phosphorylation due to disturbance in the Pi metabolism (Tripathi et al., 2007; Zhao et al., 2010). The other inorganic form, As(III), which is more toxic, is a predominant species under anaerobic conditions, and enters the root via nodulin 26-like intrinsic protein (NIP) aquaporin channels (Meharg and Jardine, 2003; Bienert et al., 2008; Ma et al., 2008). It perturbs protein functioning due to the interaction with -SH group present in many proteins (Tripathi et al., 2007; Finnegan and Chen, 2012). Thus, it is necessary to study the biological processes involved in the uptake, transport, and detoxification of such heavy metals so that effective strategies can be developed for developing plants with tolerance as well as low accumulation in plant parts (Song et al., 2010). In the past, various studies have been initiated to understand the molecular networks and processes involved in As stress response. Recently, utilizing natural variations in *Arabidopsis*, several genes and components involved in As stress tolerance and physiological responses (Chao et al., 2014; Fu et al., 2014; Sánchez-Bermejo et al., 2014) have been identified.

Phosphorous is an essential macronutrient and is critical for the plant growth and development. Phosphorous deficiency negatively affects plant growth and metabolism, and induces the expression of genes involved in inorganic Pi acquisition (Raghothama, 1999; Karthikeyan et al., 2002). In *Arabidopsis*, the high affinity Pi transporters, PHOSPHATE TRANSPORTER 1;1 (PHT1;1) and PHOSPHATE TRANSPORTER 1;4 (PHT1;4), have shown to play important role in As(V) uptake (Shin et al., 2004). Various studies suggest that the Pi starvation responses are under strict transcriptional control through various transcription factors. These transcription factors have been shown to regulate expression of PHTs and thus Pi uptake from the medium. Apart from PHT1;1 and PHT1;4 modulation, As(V) exposure also induces a notable transposon burst in the plants, which is restricted by WRKY6, thus emphasizing the importance of regulatory genes in Pi homeostasis under As(V) stress (Castrillo et al., 2013).

The general detoxification mechanism for As comprises reduced As uptake, extrusion out of the cells or sequestration of As-PC (phytochelatins) complexes inside the vacuole (Shukla et al., 2013; Shri et al., 2014). In recent years, studies have utilized *Arabidopsis* natural variations to understand the differential effect of Pi starvation on the accessions (Narang et al., 2000; Chevalier et al., 2003; Reymond et al., 2006) or their response toward As stress (Chao et al., 2014; Fu et al., 2014; Sánchez-Bermejo et al., 2014). In addition, studies suggest that Pi starvation during As exposure plays important role in its uptake and stress response (Remy et al., 2012). However, no study have been carried out to understand As(V) stress under Pi starvation using these natural variations. In the present study, the natural variations in *Arabidopsis* have been utilized to study growth response toward Pi availability and As(V) uptake at the molecular level. Study suggests differential response of selected *Arabidopsis* accessions (Col-0, Sij-1, and Slavi-1) in terms of root length under different Pi and As(V) concentrations. To get an insight into the extent of biodiversity and the identification of underlying plausible mechanisms in providing differential stress response in *Arabidopsis* natural variants, expression profiling of the genes involved in Pi/As uptake, detoxification mechanism as well as regulatory factors have been carried out. Analysis suggests differential expression of a set of genes, which might lead to differential response in *Arabidopsis* natural variations.

## Materials and Methods

### Plant Material and Growth Conditions

The seeds of three *Arabidopsis thaliana* accessions Columbia-0 (Col-0, CS60000), Sijak-1 (Sij-1, CS76379), and Slavianka-1 (Slavi-1, CS76419) were obtained from *Arabidopsis* Biological Resource Center (https://abrc.osu.edu/). The seeds were surface sterilized with 70% ethanol (v/v) for 1 min, 4% NaOCl (v/v) for 4 min, followed by washing with distilled water and were placed on 0.5X Murashige and Skoog (MS) medium (Murashige and Skoog, 1962) supplemented with 1.5 % (w/v) sucrose and 0.8 % (w/v) agar. For the Pi sufficient condition, 1.25 mM KH_2_PO_4_ was added to the medium, while for the Pi deficient condition, 15 μM KH_2_PO_4_ was used. For the Pi deficient media, KH_2_PO_4_ was replaced with KCl. For As(V) treatment, 50 μM Na_2_HAsO_4_ (Stock Solution 50 mM; Na_2_HAsO_4_, ICN, USA) was added in the media and pH was adjusted to 5.5 using 0.1 M KOH or HCl. After stratification at 4°C for 2 days, plates were transferred to a growth chamber set at 16/8 h light-dark cycle, 250 μmol m^-2^ s^-1^ light intensity and 22°C temperature.

### Evans Blue Staining

Evans blue is a non-permeating dye and is used to determine the dead cells in plant samples. *Arabidopsis* accessions were grown on Pi sufficient, Pi sufficient + As(V), Pi deficient and Pi deficient + As(V) medium for 10 days. Seedlings were incubated in Evans blue solution [0.15% (w/v) Evans blue in water] for 1 h (Dubey et al., 2014). After washing for 10 min with water, seedlings were observed for the tissue damage under Stereoscope Zoom binocular microscope (Leica S8AP0, Germany).

### RNA Isolation, cDNA Preparation, and Gene Expression Analysis

Total RNA from 10 days old seedlings was isolated using Spectrum Plant Total RNA Kit (Sigma–Aldrich, USA) as per manufacturer’s instructions. RNA was quantified using NanoDrop spectrophotometer (NanoDrop, Wilmington, DE, USA) and the quality was assessed using 1.2% agarose gel electrophoresis. Genomic DNA contamination was removed using RNase-free-DNase-I (Fermentas, Life Sciences, ON, Canada). Approximately, 1 μg total RNA was reverse transcribed using RevertAid First Strand cDNA synthesis kit (Fermentas, Life Sciences, ON, Canada) according to the manufacturer’s instructions. Quantitative real time-PCR was performed using SYBR Green Supermix (ABI Biosystems, USA) in an ABI 7500 instrument (ABI Biosystems, USA). Tubulin gene was used as an internal control to estimate the relative transcript level of the genes analyzed. The list of oligonucleotides used in the study is provided in the Supplementary Table S1. The PCR was performed in a final volume of 10 μL containing 1 μL of each of the forward and reverse primers (5 pM), 5 μL of the SYBR green master mix and 1 μL of cDNA (1:10 dilution), and 2 μL of nuclease free water. All PCR reactions were performed in the triplicate. The PCR conditions were 50°C for 2 min, 95°C for 2 min for initial denaturation followed by 40 cycles of 95°C for 15 s, and 60°C for 60 s. Data was analyzed using comparative Ct (2^-ΔΔct^) method (Schmittgen and Livak, 2008).

### Nucleotide Sequence Analysis

To analyze variations in nucleotide and deduced amino acid sequences in ACR2 gene (At5g03455) in different accessions, full-length cDNA was amplified using oligonucleotides spanning complete open reading frame (Supplementary Table S1). Amplicons were sequenced from both the ends using 96 capillary automated sequencing systems (ABI 3730 DNA Analyzer, UK).

### Arsenic Estimation

Ten days old seedlings of *Arabidopsis* accessions; Col-0, Sij-1, and Slavi-1 were thoroughly washed with distilled water and air dried for 4–5 days followed by overnight drying in an oven at 80°C. Dried samples (∼100 mg) were digested in HNO_3_ and H_2_O_2_ (3:1 v/v) at 80°C on a hot plate till the samples were converted into fine residue. The residue from digested samples was dissolved in 5 ml distilled water and filtered using filter paper (Whatman^TM^ 125 mm). The samples were used for the total As determination through an Inductively Coupled Plasma Mass Spectrometer (ICP-MS, Agilent 7500 USA) as per the standard protocol (Dubey et al., 2014). The standard reference metals (E-Merck, Germany) were used for the calibration and quality assurance for each analytical batch.

### Statistical Analysis

Each experiment was carried out under completely randomized design with three replicates repeated at least thrice. The data were analyzed by Student’s unpaired *t*-test, and the treatment mean values were compared at *P* ≤ 0.05–0.001.

## Results And Discussion

### Natural Variation in Response to Pi and As(V) Exposure

**FIGURE 1:**
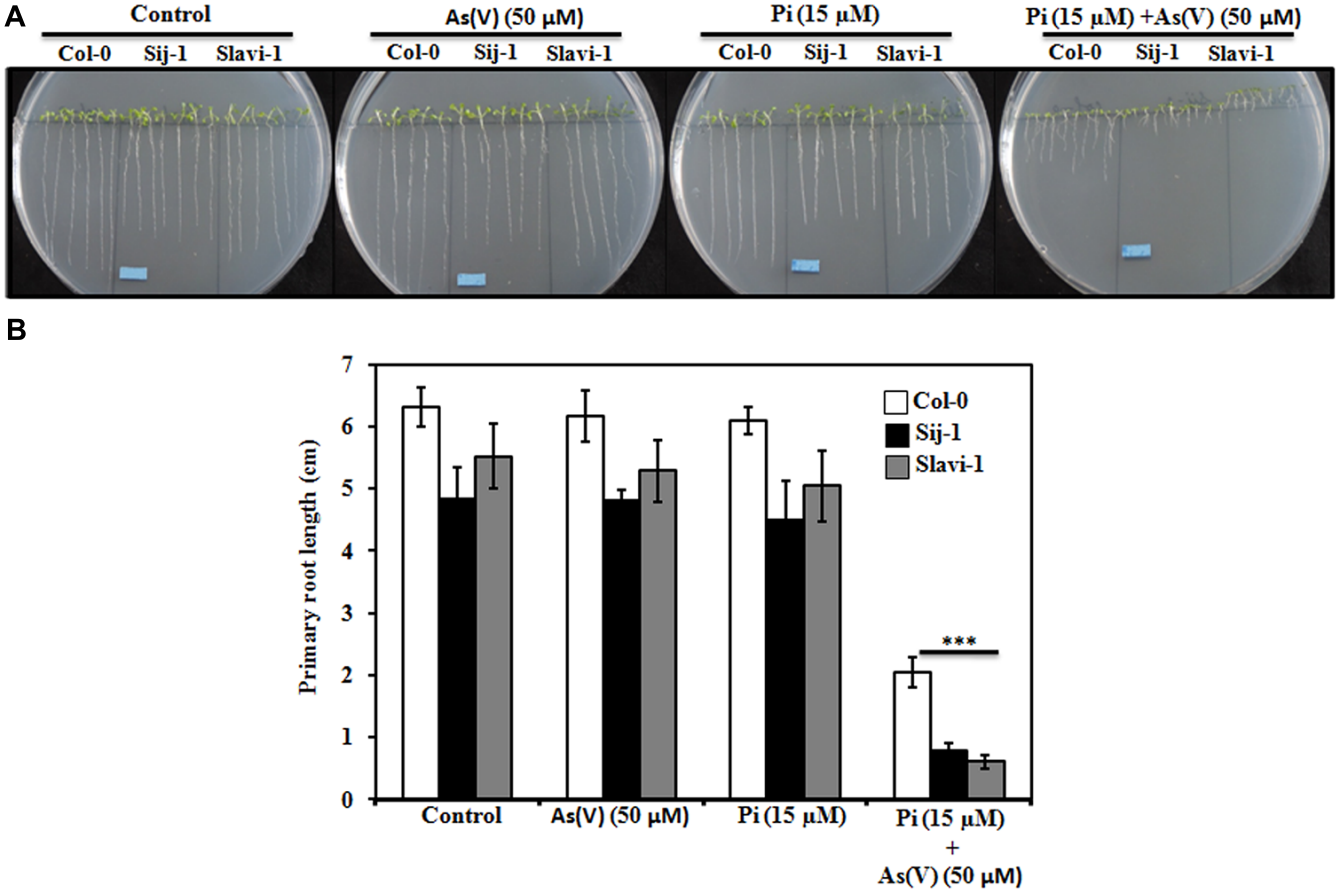
**Effect of limiting phosphate (Pi) and As(V) on the growth of *Arabidopsis* accessions.** Seeds were grown on 0.5X MS medium and the root length was evaluated after 10 days of growth. **(A)** Phenotype of *Arabidopsis* accessions on 0.5X Murashige and Skoog (MS) media (control), As(V) (50 μM), Pi (15 μM; Pi limitation), and Pi (15 μM) supplemented with As(V) (50 μM). Scale bar = 1cm. **(B)** Primary Root length of the three accessions grown under different treatments. Data are mean ± SD calculated from three biological replicates per treatment. Experiments repeated thrice with similar results. ^∗∗∗^ indicate values that differ significantly from control at *P* < 0.001, according to student’s unpaired *t*-test.

Phosphate deficiency causes a profound effect on the root morphology of the plants (Chevalier et al., 2003). Various studies have reported natural variations among *Arabidopsis* accessions in response to Pi deficiency (Narang et al., 2000; Chevalier et al., 2003; Reymond et al., 2006). Recently, natural variations for As(V) stress response has also been shown in different plant species including *Arabidopsis* (Chao et al., 2014; Fu et al., 2014; Sánchez-Bermejo et al., 2014) and rice (Rai et al., 2010; Wu et al., 2011; Sharma et al., 2015). However, no information is available for the response of these natural variants toward combined stress of Pi deficiency and As(V). Therefore, to understand the effect of genetic variations on plant growth and development, different accessions were analyzed for their response toward As(V) stress. Among different accessions Col-0, Sij-1, and Slavi-1 were identified as tolerant, moderate and sensitive, respectively, toward As(V) stress. In order to understand the interaction between As(V) and Pi uptake, these accessions were grown on optimum Pi concentration (Control; Pi-sufficient; 1.25 mM) and low Pi concentration (Pi-deficient; 15 μM). No significant change in the primary root length was observed in the three ecotypes under limiting Pi as compared to optimum Pi condition (**Figures 1A,B**).

Since it is well known that As(V) is an analog of Pi and it competes with the Pi uptake system (Raghothama, 1999; Catarecha et al., 2007; Wu et al., 2011), the root morphology was compared in the three accessions under Pi-sufficient medium supplemented with As(V) (50 μM). No significant effect on the root length was observed in the three accessions grown on Pi sufficient medium under As(V) stress (**Figures 1A,B**). However, continuous growth of *Arabidopsis* natural variants on the medium containing As(V) and Pi limiting condition caused a significant decrease in the root length. This suggests differential interaction and competition between Pi and As(V) uptake in these selected *Arabidopsis* natural variants (**Figures 1A,B**). Differential reduction in the root length was observed in the three accessions. Lesser reduction in the root length was observed in Col-0 (60%) as compared to Sij-1 and Slavi-1(>80%), (**Figures 1A,B**) suggesting better tolerance and adaptation of Col-0 under combined stress of limiting Pi and As(V). Hence, it was inferred that Col-0 is more tolerant toward Pi deficient + As(V) stress in comparison to other natural variants.

### Natural Variation in As(V) Induced Tissue Damage

**FIGURE 2:**
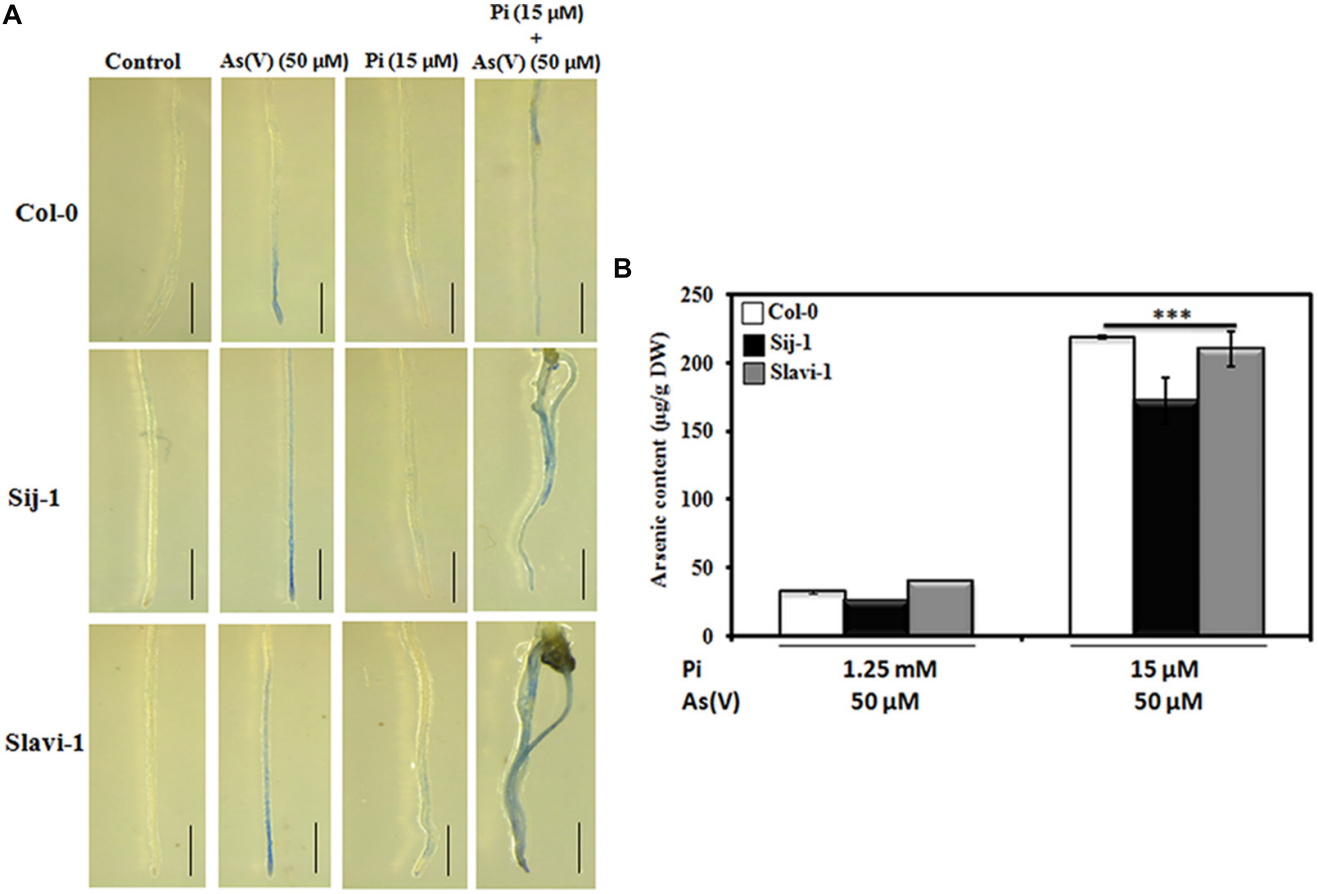
**Cell viability/death during limiting Pi and As(V) stress using Evan’s blue staining and metal accumulation by *Arabidopsis* accessions.** **(A)** Seeds of *Arabidopsis* accessions were grown for 10 days on medium containing sufficient Pi (1.25 mM) (control), As(V) (50 μM), deficient Pi (15 μM) and deficient Pi (15 μM) supplemented with As(V) (50 μM). The seedlings were incubated with Evan’s blue and observed under Stereoscope Zoom binocular microscope (Leica S8AP0, Germany) Scale bar = 1 mm. **(B)** The seeds were grown on Pi sufficient medium containing As(V) (50 μM) and deficient Pi medium (15 μM) supplemented with As(V) (50 μM) for 10 days and arsenic content was estimated using ICP-MS. Data are mean ± SD calculated from three biological replicates per treatment.^∗∗∗^ indicate values that differ significantly from control at *P* < 0.001, according to student’s unpaired *t*-test.

As(V) induces the production of reactive oxygen species (ROS) inside the plant cell leading to lipid peroxidation and damage to proteins and nucleic acids (Halliwell, 2006; Møller et al., 2007). The induced oxidative stress during As(V) stress is combated by antioxidant enzymes in concert with non-enzymatic antioxidants such as Non-Protein Thiols (NPTs) and glutathione (GSH; Shri et al., 2009; Rai et al., 2010). It has been reported that the increased production of ROS results into cellular damage and ultimately cell death (Breusegem and Dat, 2006). Therefore, As(V) induced cellular damage was analyzed in the accessions using Evans Blue staining. It was observed that in comparison to control (sufficient Pi) condition, under Pi sufficient + As(V) and Pi deficient + As(V) conditions, Sij-1 and Slavi-1 were severely affected by As(V) stress as compared to Col-0 (**Figure 2A**). The cellular damage was indicated by deeply stained tissues in these natural variants. This is in corroboration with the phenotypic analysis, which showed that the impact of As(V) stress was more pronounced in Sij-1 and Slavi-1 as compared to Col-0 (**Figures 1A,B**). Also, our study is in corroboration with the studies carried out on the rice root, where differential staining pattern was observed for different heavy metals, which is an indicator of varying degree of toxicity caused by heavy metals upon their accumulation (Dubey et al., 2014). Thus, Evans blue staining suggests that the toxicity and cell death due to As(V) exposure varies substantially among *Arabidopsis* accessions. Therefore, it can be inferred that *Arabidopsis* natural variants possess distinct molecular mechanisms for the acquisition of Pi as well as to sustain growth and development under As(V) stress conditions.

### Natural Variation in Arsenic Accumulation in *Arabidopsis*

Investigation of the molecular function of the genes responsible for As uptake, accumulation and metabolism is prerequisite to minimize As stress in plants (Kumar et al., 2015). Studies have reported that rice accessions differ in their As accumulation potential and are categorized as high and low As accumulating germplasms (Rai et al., 2010; Sharma et al., 2015). Therefore, to investigate the effect of Pi deficiency on As(V) uptake and accumulation, Col-0, Sij-1, and Slavi-1 were grown on Pi sufficient and deficient medium supplemented with As(V). It was observed that As accumulation potential was equivalent in all the accessions under both Pi sufficient and deficient conditions. However, under Pi deficient + As(V) condition, accessions accumulate many folds higher As compared to Pi sufficient + As(V) condition due to enhanced As uptake (**Figure 2B**). Similar observation with enhanced As uptake under Pi deficiency has been observed in *Arabidopsis* and rice (Catarecha et al., 2007; Dubey et al., 2014). Thus, it can be inferred that with decreasing Pi concentration As accumulation increases, however, accumulation potential does not differ significantly between different natural variants in these accessions. This suggests that differential As(V) response in these natural variants might be dependent on detoxification mechanism involving transport, accumulation or regulatory mechanisms.

### Differential Expression of Genes Related to Transport System

**FIGURE 3:**
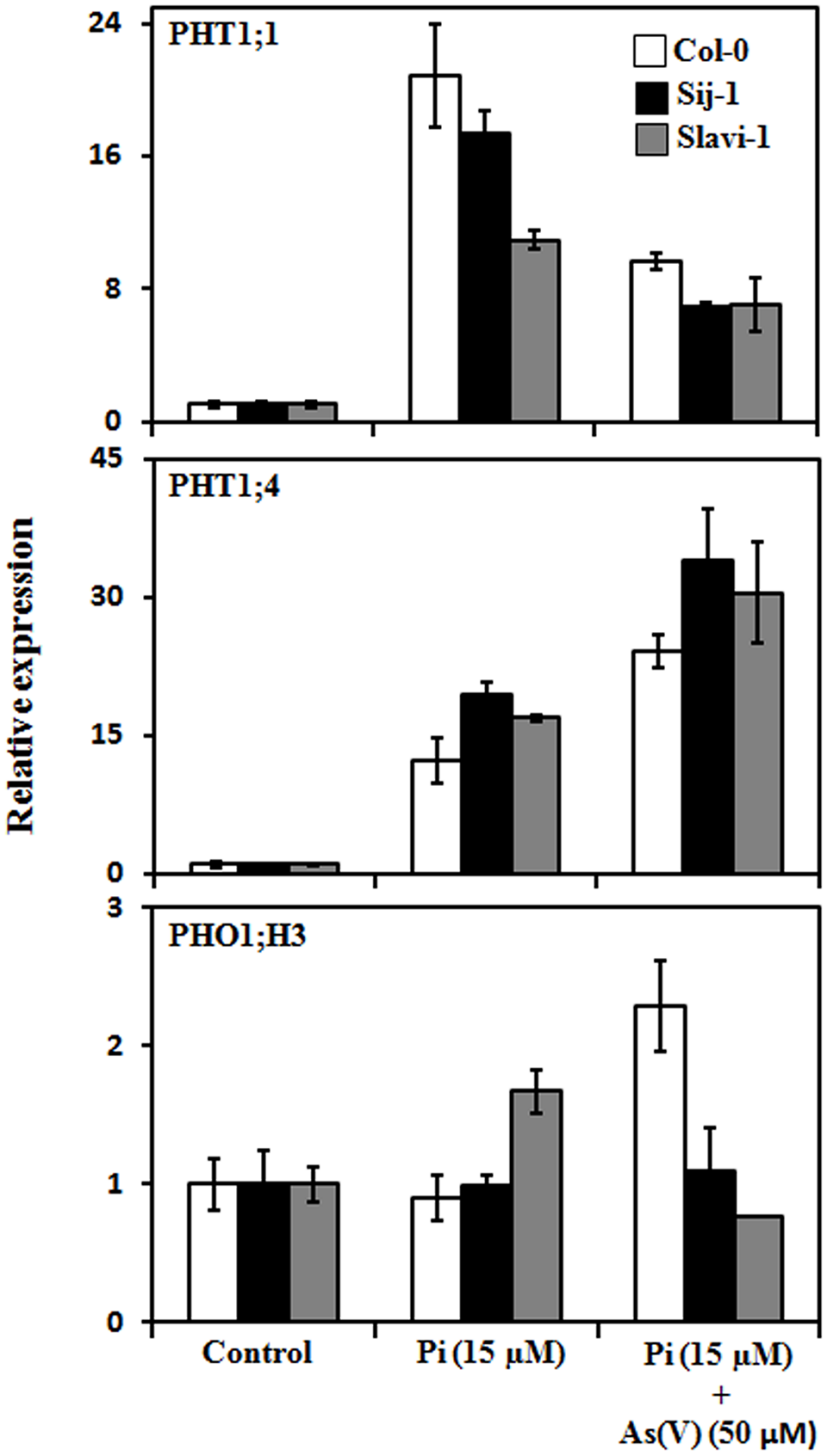
**Expression analysis of genes involved in Pi and As(V) uptake under sufficient Pi (1.25 mM) (control), deficient Pi (15 μM), and deficient Pi (15 μM) supplemented with As(V) (50 μM) using real time PCR.** Error bars represent ±SD from three technical replicates of 30 seedlings each.

Phosphate enters into the plant cell via a set of Pi transporters both under Pi sufficient and deficient conditions (Shin et al., 2004). PHT1;1 and PHT1;4 are high affinity Pi transporters, which expresses in the root epidermis and root hair and have maximum transcript abundance among all the nine putative members of phosphate transporter (PHT) family (Shin et al., 2004; Lapis-Gaza et al., 2014). In *Arabidopsis*, among different members of Pi transporters, PHT1;1 and PHT1;4 are known to play an important role in As(V) uptake (Shin et al., 2004). To understand the genetic variations with respect to expression of PHTs, the transcript abundance of these two Pi transporters was analyzed in the seedlings of Col-0, Sij-1, and Slavi-1. Differential expression pattern of PHT1;1 was observed in all the accessions with enhanced expression of PHT1;1 under Pi deficiency in comparison to control (Pi sufficient condition; **Figure 3**). Under limiting Pi, PHT1;1 expression was highest in Col-0 followed by Sij-1 and Slavi-1 (**Figure 3**). The expression pattern of PHT1;1 in the presence of As(V) decreased significantly as compared to limiting Pi condition in the natural variants. This decrease in the expression of PHT1;1 was similar in Col-0 and Sij-1 (∼50%); however, lesser change in expression was observed in Slavi-1 (∼25%). Similar to PHT1;1, the expression of PHT1;4 was higher under Pi deficiency in comparison to control in all the *Arabidopsis* natural variants. Conversely, the expression of PHT1;4 was lower in Col-0 in comparison to other accessions in both Pi deficient and Pi deficient + As(V) conditions (**Figure 3**). This suggests differential regulation of these PHTs during limiting Pi and in the presence of As(V). As it is already known that regulatory factors responsible for the expression of both these PHTs during Pi deficient conditions are different (Devaiah et al., 2007a,b; Nilsson et al., 2007; Duan et al., 2008; Karthikeyan et al., 2009; Castrillo et al., 2013; Wang et al., 2014), it seems that differential regulation by transcription factors might be responsible for the natural variation in *Arabidopsis* in response to nutrient deficiency and As(V) stress.

Further, in *Arabidopsis*, Phosphate transporter 1 (PHO1) has been identified to be involved in the loading of inorganic Pi into the xylem of roots (Hamburger et al., 2002). PHO1 mainly expresses in the root cells and helps in the maintenance of Pi homeostasis (Hamburger et al., 2002). PHO1 homolog, PHO1;H3 is up regulated under Zn-deficiency and negatively regulates Pi loading into the xylem of root tissues (Kisko et al., 2014). Since PHO1;H3 is known to have a major role in crosstalk between heavy metal Zn and Pi under Zn deficiency conditions (Khan et al., 2014), it was analyzed that whether the expression pattern of PHO1;H3 was also modulated under Pi-deficient and Pi-deficient + As(V) stress in *Arabidopsis* natural variants. Differential expression of PHO1;H3 was observed in Col-0 and Slavi-1, whereas no modulation in the expression was observed in Sij-1 under both Pi-deficient and Pi-deficient + As(V) conditions (**Figure 3**). Under Pi deficiency, increased PHO1;H3 expression in Slavi-1 suggests less Pi mobilization in comparison to Col-0 and Sij-1 as it is a negative regulator of Pi movement via xylem. The decreased expression of PHO1;H3 in Slavi-1 under Pi deficient + As(V) condition results into increased Pi movement, which might lead to enhanced As(V) translocation causing hampered growth and sensitivity. Under Pi deficiency, no modulation in the PHO1;H3 expression was observed in Col-0 suggesting better Pi mobilization toward the shoot via xylem, whereas, enhanced expression of PHO1;H3 was observed in Col-0 under Pi deficient + As(V) condition (**Figure 3**) further suggests restricted Pi movement and so as that of As(V). Intriguingly, in spite of severe growth retardation, no significant modulation in the expression of PHO1;H3 was observed in Sij-1. Therefore, the differential expression pattern of PHTs and PHO1;H3 under Pi deficient and Pi deficient + As(V) conditions suggests that although these transporters are involved in Pi acquisition from soil and its homeostasis, their expression may be differentially regulated in *Arabidopsis* accessions.

### Transcription Factors and Natural Variations under Low Pi and As(V)

Previous reports have accounted the role of transcription factors in regulating Pi starvation responses in plants (Wu et al., 2003; Misson et al., 2005). Therefore, we analyzed the expression pattern of WRKY and other Pi and As(V) responsive transcription factors in *Arabidopsis* accessions under Pi deficiency and Pi deficient + As(V) conditions.

#### Modulation in the Expression of WRKY Transcription Factors

**FIGURE 4:**
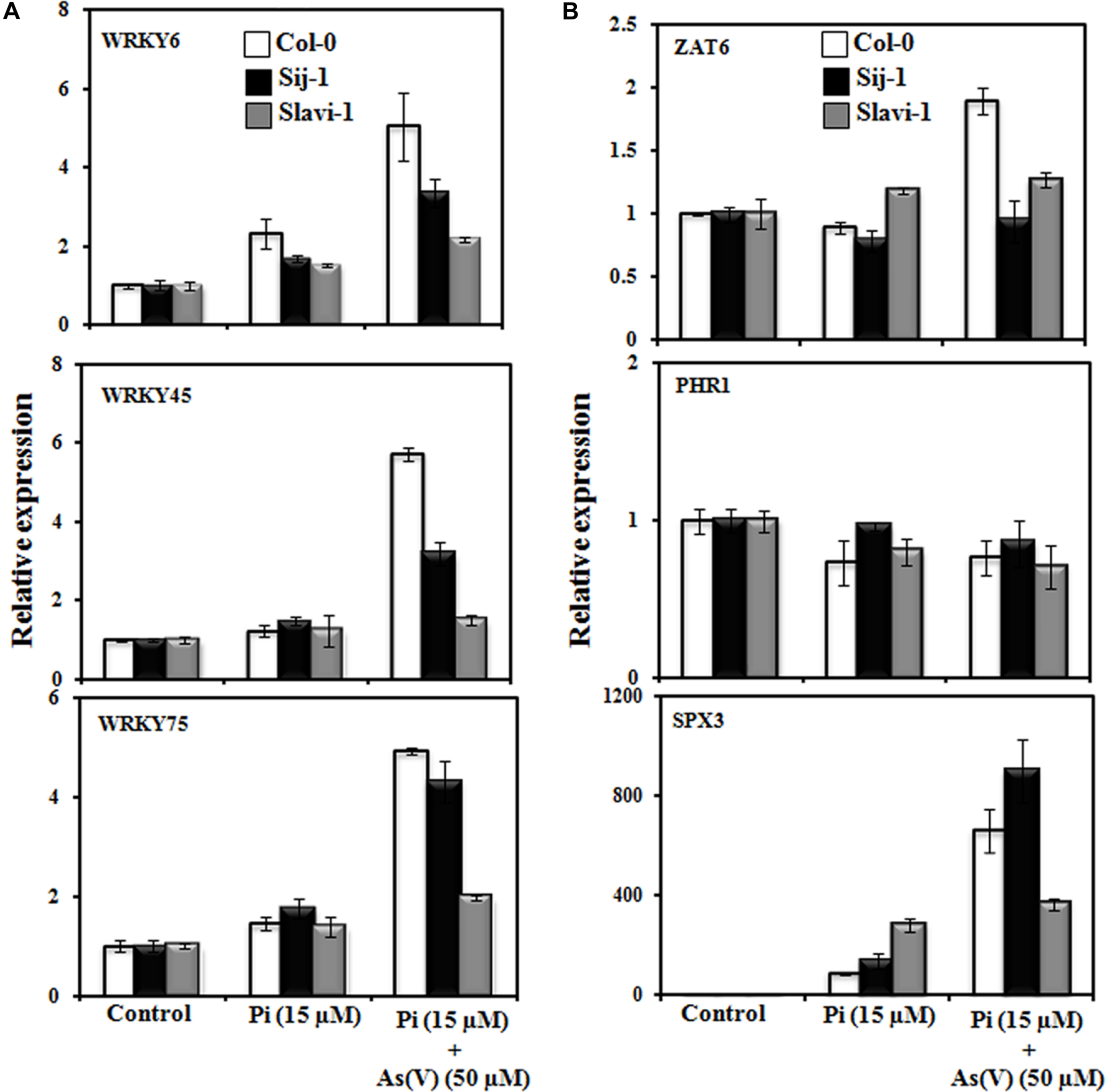
**Transcription factors mediated response of *Arabidopsis* accessions under Pi deficient and As(V) stress.** **(A)** Expression analysis of members of WRKY transcription factor gene family and **(B)** Other transcription factors known to regulate Pi homeostasis. Error bars represent ±SD from three technical replicates of 30 seedlings each.

WRKY6 is an As(V)-responsive transcription factor, which negatively regulates PHT1;1 expression (Castrillo et al., 2013) and has role in defense against other stresses (Robatzek and Somssich, 2002). Expression analysis suggests differential expression pattern of WRKY6 in the *Arabidopsis* accessions; with a maximum expression in Col-0 (fivefold) followed by Sij-1 (>3-fold) and Slavi-1 (>2-fold), under Pi-deficient + As(V) condition in comparison to Pi deficient condition (**Figure 4A**). As WRKY6 is known to repress the expression of PHT1;1, in the presence of As(V) as described by Castrillo et al. (2013), this can be easily correlated with the increased WRKY6 expression (**Figure 4A**) and decreased PHT1;1 expression (**Figure 3**) in all the accessions under Pi deficient + As(V) stress. Earlier report by Catarecha et al. (2007) has shown that expression of PHT1;1 is significantly down regulated under As(V) stress and this is in correlation with our data that activation of WRKY6 in response to As(V) stress might reduce the expression of PHT1;1 and thus As(V) toxicity (**Figures 3** and **4A**).

WRKY45 is known to be induced under Pi starvation, and recently, WRKY45 was reported to positively regulate the expression of PHT1;1 but not PHT1;4 (Wang et al., 2014). Interestingly, the expression of WRKY45 was not significantly induced upon Pi deficiency in all the three accessions (**Figure 4A**), but the expression of PHT1;1 significantly increased in Col-0 (∼20-fold), Sij-1 (∼15-fold) and Slavi-1 (∼10-fold), (**Figure 3**). Our analysis showed that WRKY45 expression is also induced under Pi-deficient + As(V) condition (**Figure 4A**) but this induction did not modulate the expression of PHT1;1 (**Figure 3**). This suggests a different regulatory mechanism of PHT1;1 regulation under Pi-deficient + As(V) condition, which might be mediated by transcription factors other than WRKY45.

In a recent study, WRKY75 was demonstrated to regulate Pi homeostasis by controlling both the Pi acquisition and modulation in the root architecture (Devaiah et al., 2007a). It was observed that the expression of WRKY75 is induced under Pi-deficiency (**Figure 4A**) which might have resulted into strong induction of PHT1;1 expression under the same condition (**Figure 3**). Enhanced expression of WRKY75 was also observed in all the accessions in Pi deficient + As(V) stress with a maximum increase in Col-0 (fivefold) followed by Sij-1 (4.4-fold) and Slavi-1 (twofold) as compared to that in Pi deficient condition (**Figure 4A**). But this induction in WRKY75 expression had no effect on modulating the expression of PHT1;1 under Pi deficient + As(V) stress condition, similar to that of WRKY45 (**Figures 3** and **4A**). Altogether, the analysis suggests that WRKY45 and WRKY75 positively regulate the expression of PHT1;1, which might lead to increased expression of PHT1;1 under Pi-deficiency, in spite of the similar metal accumulation potential of the accessions (**Figure 2B**). The increased expression in Col-0 in comparison to Sij-1 and Slavi-1 suggested better Pi acquisition potential of Col-0 from the external medium.

The expression analysis under Pi-deficient + As(V) stress revealed that while the expression of WRKY45 and WRKY75 was induced in the presence of As(V), the expression of PHT1;1 decreased (**Figures 3** and **4A**). This down regulation in PHT1;1 expression might be due to WRKY6, which plays important role in rescue mechanism of plants to avoid As toxicity (Castrillo et al., 2013). In spite of the down regulation of PHT1;1 under Pi-deficient + As(V) condition and similar metal accumulation potential as that of Col-0, the accessions Sij-1 and Slavi-1 were severely affected by As(V) toxicity, the probable reason could be the functional redundancy of PHT transporters. Expression of most of these transporters are regulated by different transcription factors, therefore, although PHT1;1 is sufficiently repressed putatively by WRKY6; these accessions still suffered severe toxicity.

#### Modulation in Expression of ZAT6 Transcription Factor

Expression analysis has revealed that significant enhanced expression of ZAT6 is observed only in Col-0 under Pi deficient + As(V) condition as compared to that in Pi deficient condition (**Figure 4B**). Previous study by Devaiah et al. (2007b) suggested that ZAT6 negatively regulates the expression of PHT1;1, therefore, we analyzed the correlation between ZAT6 expression and PHT1;1 repression under Pi deficient and Pi deficient + As(V) condition in *Arabidopsis* natural variants (**Figure 4B**). Results demonstrated that ZAT6 expression is not significantly modulated, however; PHT1;1 expression was differentially enhanced under Pi-deficient condition as compared to control (**Figures 3** and **4B**). It was observed that PHT1;1 expression decreased in all the accessions under Pi deficient + As(V) condition (**Figure 3**) but no significant change in ZAT6 expression was observed under same condition (**Figure 4B**) except in Col-0. Differential modulation in ZAT6 expression in natural variants and specific enhancement in Col-0 might be regulating PHT1;1 as well as metal stress response. However, the exact role of ZAT6 in As(V) stress response needs to be functionally validated.

#### Modulation in Expression of PHR1 Transcription Factor

Studies on phr1 mutants and overexpressing lines emphasized that PHOSPHATE STARVATION RESPONSE 1 (PHR1) is a central regulator of Pi starvation responses (Nilsson et al., 2007; Bustos et al., 2010). It play important role in plant development under different stress conditions (Rubio et al., 2001; Rouached et al., 2011; Nilsson et al., 2012) and also participates in long distance Pi signaling in plants (Bari et al., 2006; Lin and Chiou, 2008). It regulates Pi homeostasis by binding to P1BS motifs present in the promoter region of the genes, which are regulated (Rubio et al., 2001; Franco-Zorrilla et al., 2004; Stefanovic et al., 2007). Thus, we analyzed its expression under Pi deficiency and Pi deficient + As(V) condition. Result suggests that expression of PHR1 did not significantly modulate in response to Pi deficiency and Pi deficiency + As(V) condition in any of the accessions, which was in accord with the other studies (**Figure 4B**), (Rubio et al., 2001). It seems that post-translational modifications of PHR1 as demonstrated earlier (Miura et al., 2005) might be responsible for the differential Pi and As(V) response in *Arabidopsis* natural variants.

#### Modulation in the Expression of SPX Transcription Factor

It has been reported that Pi deficiency induces the expression of AtSPX3 (Shi et al., 2014) which plays important role in restoring Pi balance following Pi starvation (Duan et al., 2008). Therefore, the expression pattern of *AtSPX3* was investigated in natural variations in response to different treatments including Pi sufficient, Pi deficient and Pi deficient + As(V) stress conditions. In response to Pi deficiency, enhanced expression of AtSPX3 was observed in Slavi-1 (300-fold) and Sij-1 (>100-fold) in comparison to Col-0 (∼100-fold) as compared to control condition (**Figure 4B**). Previous studies suggest that AtSPX3 is induced by PHR1 and exerts negative feedback control over AtSPX1, which is involved in the regulation of various genes encoding regulatory enzymes such as RNS1 (Pi remobilization), PAP2 (anthocyanin biosynthesis), IPS1; At4 (Pi allocation), PHT1;4 and PHT1;5 (Pi transport) to circumvent Pi induced hypersensitive responses during prolonged Pi starvation (Duan et al., 2008). Our result also demonstrated that in Col-0 the expression of PHT1;4 was lower as compared to Sij-1 and Slavi-1 (**Figure 3**) which reflect that these accessions might require more Pi through PHT1;4 under Pi deficient condition. The expression of AtSPX3 was also evaluated under Pi-deficient + As(V) stress. Interestingly, significantly enhanced expression was observed in Sij-1 (>900-fold), Col-0 (>600-fold) and Slavi-1 (>300-fold) as compared to Pi deficient condition. The observed expression pattern during Pi deficiency was altered with the supplementation of As(V) and least expression was observed in Slavi-1 whilst Sij-1 showed highest expression (**Figure 4B**). Similar expression pattern was observed for PHT1;4 under Pi-deficient + As(V) stress as compared to Pi deficient condition (**Figure 3**) suggesting that though SPX3 exerts a negative regulation over PHT1;4 under Pi starvation, its repression is altered in the presence of As(V) which might result in the enhanced expression of PHT1;4 in Sij-1 and Slavi-1 causing increased toxicity in these accessions in comparison to Col-0.

### Natural Variation in the Expression of Genes Involved in Detoxification System

In order to combat As stress, plant should possess an efficient detoxification system (Tripathi et al., 2012; Kumar et al., 2013a,b). It is well documented that As(V) after entering into the plant cell via high affinity Pi transporters is converted to As(III) by arsenate reductase, which is another inorganic and more toxic form of As (Dhankher et al., 2006; Chao et al., 2014; Sánchez-Bermejo et al., 2014). Recently, natural variation in As(V) tolerance identified a quantitative trait locus encoding arsenate reductase (ACR2; Sánchez-Bermejo et al., 2014). In a different study, GWA mapping identified the same locus involved in controlling variation in As accumulation in plants termed as High Arsenic Content 1 (HAC1), which is an arsenate reductase required to reduce As(V) to As(III) (Chao et al., 2014). However, in our study, modulation in the expression of ACR2 was not observed in the three accessions (Supplementary Figure S1). In addition, the nucleotide sequencing of ACR2 in the three accessions was carried out, which showed no difference in the nucleotide sequence and thus in the protein encoded by ACR2 in Col-0, Sij-1, and Slavi-1 (Supplementary Figure S2). As(V) tolerance is usually linked with the slow As(V) uptake (Meharg and Macnair, 1991, 1992), and increase As accumulation (Catarecha et al., 2007; Castrillo et al., 2013). As a detoxification mechanism, As(V) is reduced to As(III), which is subsequently sequestered inside the vacuole as phytochelatins (PCs)-metal complex through tonoplast localized AtABCC1 and AtABCC2 transporters (Briat, 2010; Song et al., 2010).

**FIGURE 5:**
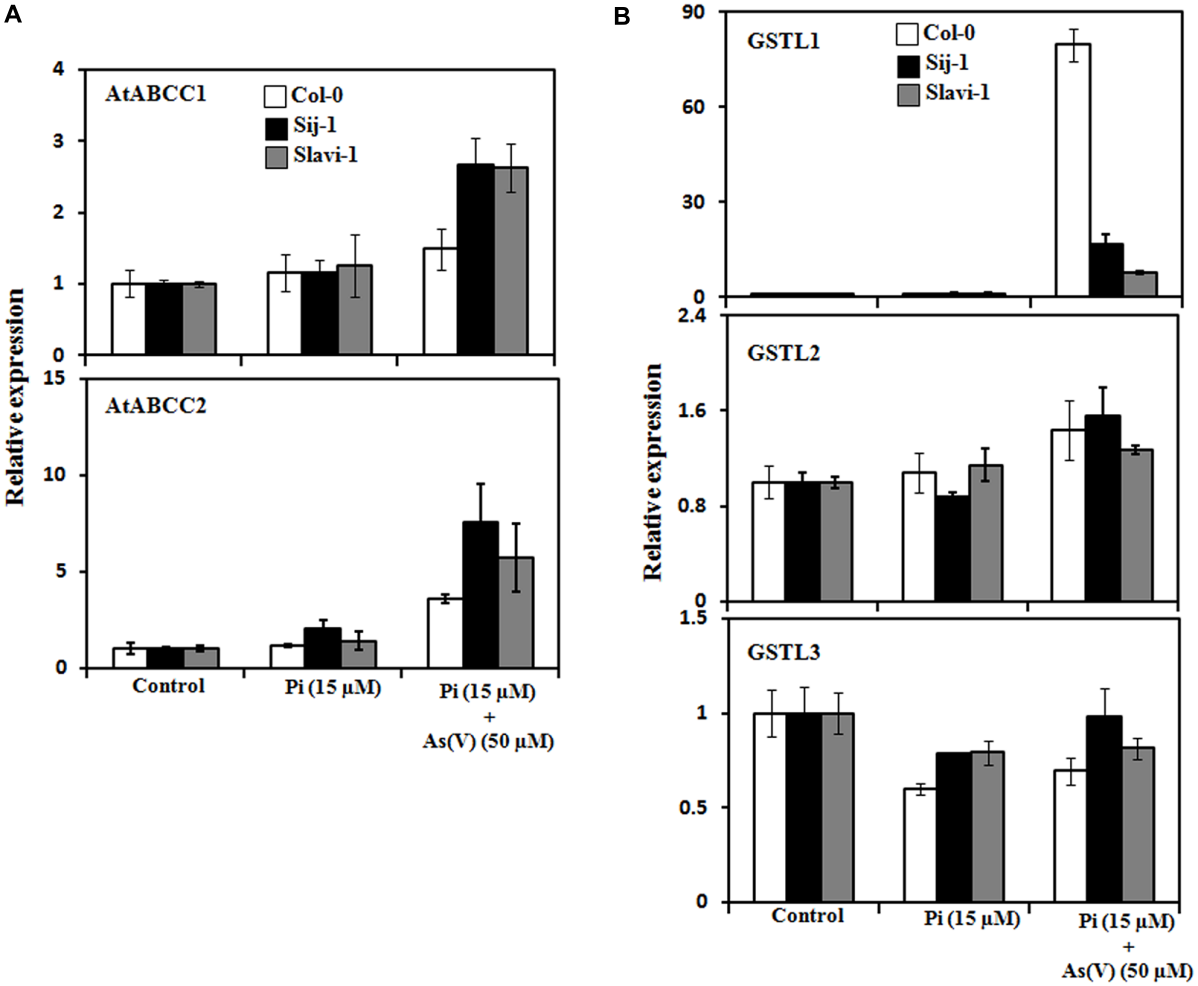
**Modulation in the expression of genes involved in detoxification mechanism in *Arabidopsis* accessions under limiting Pi and As(V) stress.** **(A)** qRT-PCR analysis of transporters involved in sequestration of PC-As(III) complexes inside the vacuole. **(B)** qRT-PCR of AtGSTL members involved in As detoxification. Error bars represent ±SD from three technical replicates of 30 seedlings each.

In order to have an insight into the modulation in the expression of these transporters and the genetic variations occurring in response to As stress and Pi deficiency, the expression pattern was analyzed in all the three selected accessions grown under different experimental conditions. Significant up-regulation of the genes encoding AtABCC1 and AtABCC2 were observed in Sij-1 and Slavi-1 in comparison to Col-0 under Pi deficient + As(V) stress as compared to Pi sufficient condition (**Figure 5A**). This suggests that Sij-1 and Slavi-1 may accumulate more As, resulting increased sensitivity toward As stress. However, metal accumulation in Col-0, Sij-1, and Slavi-1 under Pi sufficient + As(V) stress demonstrated no significant variation in As accumulation in all the accessions. Increased level of As accumulation was observed in Col-0 (∼sevenfold) in comparison to Slavi-1 and Sij-1 (∼fivefold) under Pi deficient + As(V) as compared to Pi sufficient + As(V) stress (**Figure 2B**). Therefore, the analysis suggested that As accumulation increased under Pi starvation in three accessions at varying level. Among the three accessions, increased tolerance of Col-0 under Pi deficient + As(V) stress and also enhanced As accumulation suggests the presence of a different mechanism of detoxification conferring tolerance in Col-0 as compared to other accessions.

To understand better tolerance and adaptability of Col-0 with respect to other accessions, expression pattern of the three members of Lambda class glutathione S-transferse (GST) gene family was analyzed in natural variants exposed to different growth conditions. GSTs are a superfamily of enzymes that have a role in detoxification of xenobiotics (Dixon et al., 2002; Theodoulou et al., 2003). Recently, the role of rice Lambda class of GSTs was explored in heavy metal stress tolerance (Kumar et al., 2013a,b). The Lambda class of AtGSTs comprises of three members and out of the these members, differential expression pattern of only one member (AtGSTL1) was observed in all the accessions under Pi deficient + As(V) stress. Interestingly, a most remarkable increase in the expression of AtGSTL1 was observed in Col-0 (>80-fold) followed by Sij-1 (>10-fold), and Slavi-1 (>5-fold), (**Figure 5B**). This suggests strong detoxification machinery of Col-0 in comparison to Sij-1 and Slavi-1 and one of the possible reasons for providing tolerance to Col-0 against stress conditions in comparison to other natural variants.

**FIGURE 6:**
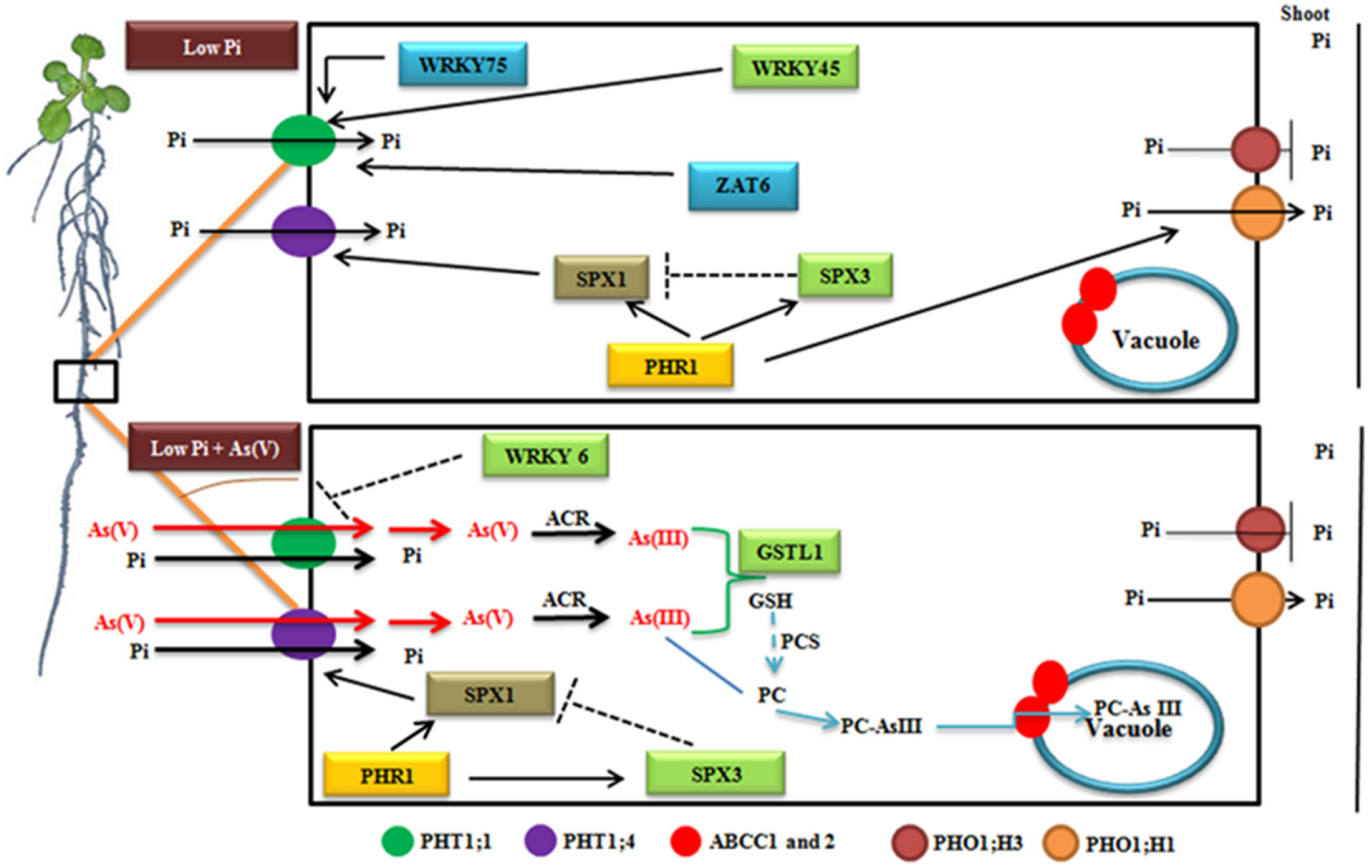
**Schematic model representing the events occurring inside the plant under low Pi and low Pi + As(V) growth conditions.** Solid lines indicate positive regulation whereas negative regulation is indicated via flattened lines.

Through expression analysis of genes in *Arabidopsis* accessions in response to low Pi and low Pi + As(V), we propose a model, which depicts the putative sequence of events occurring under these conditions (**Figure 6**). In low Pi condition, WRKY45, ZAT6, and WRKY75 positively induces the expression of PHT1;1 to acquire Pi from the medium. The expression of PHT1;4 is regulated by PHR1, which is a central regulator of Pi starvation response. During Pi starvation, PHR1 is needed for inducing the expression of SPX1 and SPX3. SPX1 positively modulates the expression of PHT1;4 whereas SPX3 exerts a negative feedback regulation over SPX1, which is prerequisite to avoid hypersensitive response during prolonged Pi starvation. After being taken up by the Pi transporters, Pi is mobilized to shoot via PHR1 induced PHO1;H1 whereas PHO1;H3 negatively regulate Pi movement. Under low Pi + As(V) stress, As(V) and Pi competes to enter inside the plant via PHT transporters. WRKY6, an As(V) responsive transcription factor, negatively regulate the expression of PHT1;1, restricting As(V) and Pi movement. PHR1 strongly induces the expression of SPX1 and SPX3 but the negative regulation of SPX3 over SPX1 is diminished resulting in increased expression of PHT1;4 and As(V) movement inside the plant. Further, As(V) is reduced to As(III) by ACR2, which is further detoxified by GSTL1 or gets sequestered inside the vacuole via ABCC1 and ABCC2 transporters (**Figure 6**).

## Conclusion

Plants evolve and adapt to plethora of environmental stresses and leads to intraspecific variations. Despite the considerable variation, little is known about the genetic basis of *Arabidopsis* response to nutrient deficiency and heavy metal stress. Our study demonstrate natural variation in *Arabidopsis* under Pi deficiency and Pi deficient + As(V) stress. Result suggest substantial contrast in the accessions (Col-0, Sij-1, and Slavi-1) toward low Pi and As(V) stress. The phenotypic data and the expression profiling of the genes involved in Pi/As(V) uptake, Pi mobilization, As detoxification and the members of different transcription factors gene family was evaluated. Out of the three accessions studied, Col-0 showed least reduction in the primary root length in comparison to other natural variants under Pi deficient + As(V) stress. In spite of the difference in the response to As(V) stress, no significant change in the capacity of metal accumulation was observed in the accessions. Expression analysis suggested a significant differential expression of PHT1;1 and PHT1;4 in three accessions, which might be the possible reason of tolerance of Col-0 toward As(V) stress in comparison to other accessions. In addition, the increased expression of AtGSTL1 and decreased expression of AtABCC1 and AtABCC2 in Col-0 as compared to Sij-1 and Slavi-1 might be responsible for better detoxification system to combat As(V) stress under Pi deficient condition. In addition, modulated expression of regulatory genes such as WRKY6 and SPX3 in different natural variants might be involved in different response of accessions to As(V) stress under Pi deficient condition. Further, the detailed analysis under combined stress conditions utilizing natural variations will help in understanding the biological processes involved in heavy metal uptake, transport and detoxification.

## Conflict of Interest Statement

The authors declare that the research was conducted in the absence of any commercial or financial relationships that could be construed as a potential conflict of interest.
