# Supplementary material for: Natural variations in expression of regulatory and detoxification related genes under limiting phosphate and arsenate stress in Arabidopsis thaliana
Source: Front Plant Sci. 2015 Oct 23;6:898. doi: 10.3389/fpls.2015.00898 (PMC4617098; doi:10.3389/fpls.2015.00898)
Supplement: Supplementary file 1 [file Data_Sheet_1.PDF]

Supplementary Information

Natural variations in expression of regulatory and detoxification related genes under limiting phosphate and arsenate stress in *Arabidopsis thaliana*

Tapsi Shukla[1, 2], Smita Kumar[3], Ria Khare[1, 2], Rudra Deo Tripathi[1, 2], Prabodh Kumar Trivedi[1, 2]

[1]CSIR-National Botanical Research Institute, Council of Scientific and Industrial Research (CSIR-NBRI), Rana Pratap Marg, Lucknow-226001, INDIA

[2]Academy of Scientific and Innovative Research (AcSIR), Anusandhan Bhawan, 2 Rafi Marg, New Delhi-110 001, India

[3]Department of Biochemistry, University of Lucknow, Lucknow 226007, India

Correspondence:

Prabodh Kumar Trivedi,
CSIR-National Botanical Research Institute (CSIR-NBRI),
Rana Pratap Marg,
Lucknow-226001, INDIA
Tel: 91-522- 2297958
Fax: 91-522-2205836, 2205839
Email: prabodht@hotmail.com; prabodht@nbri.res.in

Running title: Arabidopsis natural variations and low Pi/arsenate stress response

Supplementary Table S1: List of oligonucleotides used in this study

S.No	Name	Forward primer (5-3')	Reverse primer(3'-5')
1	PHT1;1	CCTCAACTCTCCAGAGAAGTTCTTA	TTCGGCCATTTCCTAGAGC
2	PHT1;4	CCTCTCTCTTTCTACACTCTTCTGA	CCCTTGCCATTCTTCTTCTCCT
3	TUBULIN	GAGCCTTACAACGCTACTCTGTCTGTC	ACACCAGACATAGTAGCAGAAATCAAG
4	PHO1;H3	CCTCGCAGCTCCACTTTACA	ACTCGATGCTTCGAATCGCT
5	AtABCC1	CCGCAGAAATCCTCTTGGTCTTGATG	GTGAATCATCACCGTTAGCTTCTCTGG
6	AtABCC2	AGCGTGCCAAAGATGACTCACACCAC	TACTTATCACGAAGAACACAACAGGG
7	AtTUBULIN8	CTCACAGTCCCGGAGCTGACAC	GCTTCAGTGAACTCCATCTCGT
8	GSTL1	ATGCTACCTCTGATCCACCTGC	TGGATTACAAGAAACCTCAAG
9	GSTL2	AGTTAGCGCGTTCTTACATCC	CGGGAGACGGTGGTGTAAATC
10	GSTL3	GCTCCGCTTGATGCTACTTCTG	TGGATCACTCGAAACTTCAAG
11	WRKY6	GCAACAGCAACAACAGAACAA	ACACATCCTCGGCCTCAC
12	WRKY45	TGCACAGAAGAAGGATGCAG	TGGTATGTCGTCACCACCAC
13	WRKY75	GTGGACCAAGAAGTGGTCGT	CGGTGGATTTCTCGATGGGA
14	ZAT6	ACGAAGGAAAGAACGGAGGC	ATTCCGGTATCGGCGGTATG
15	PHR1	GCAATAACGGAACGGGCAAG	AGCTCTTTCACTACCGCCAA
16	SPX3	GGGATACGAGAGATGTGCGG	TAGCGGCGGAAGTGAGAATG
17.	ACR2Full	GGTGTTTGGAGAGATATGGGGAG	TGGTTTAGTAGGGTTTAGGCGCA
18.	ACR2RT	TTCATCGTCGTCCCAACATCGCC	CGACTCGCATAGTGTAGCG

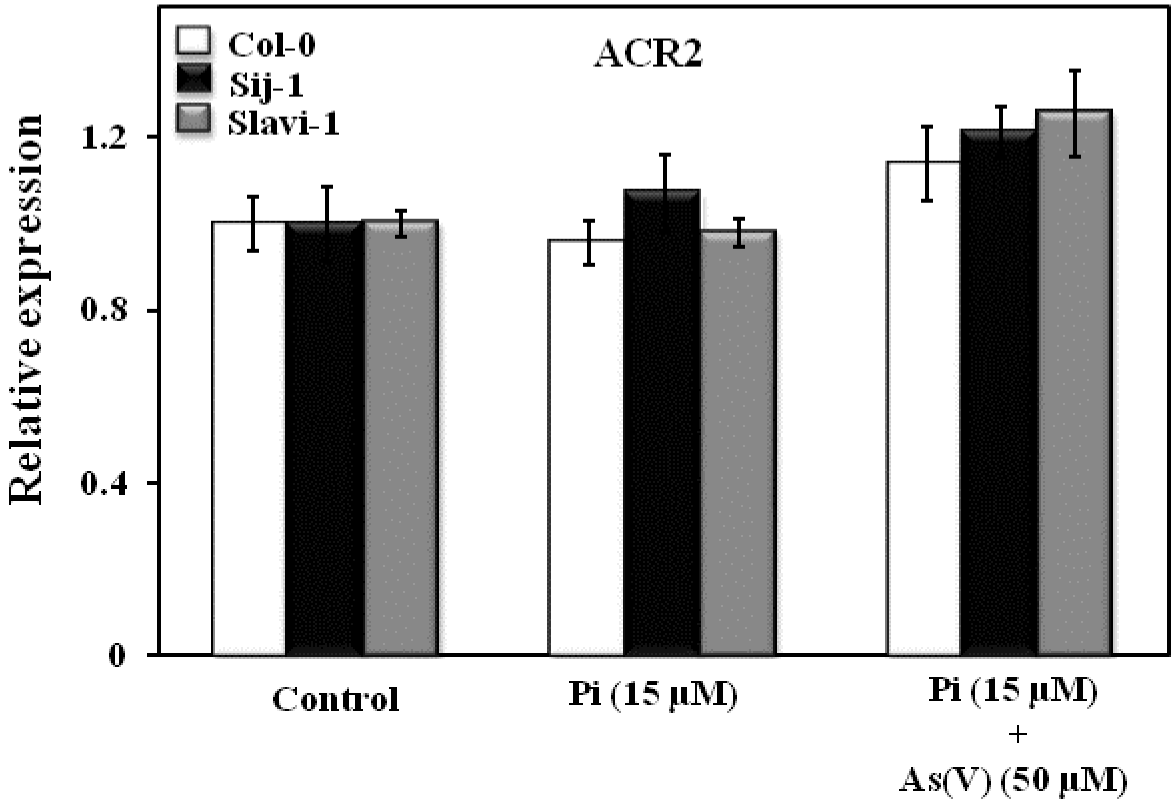

Supplementary Figure 1: Expression analysis of AtACR2 in different *Arabidopsis* accessions in response to low Pi and As(V) stress.

```
           1         10        20        30        40        50        60        70   75
           |--------+---------+---------+---------+---------+---------+---------+----|
    Col-0  MGRSIFSFFTKKKKMAMARSISYITSTQLLPLHRRPNIAIIDVRDEERNYDGHIAGSLHYASGSFDDKISHLVQN
    Sij-1  MGRSIFSFFTKKKKMAMARSISYITSTQLLPLHRRPNIAIIDVRDEERNYDGHIAGSLHYASGSFDDKISHLVQN
  Slavi-1  MGRSIFSFFTKKKKMAMARSISYITSTQLLPLHRRPNIAIIDVRDEERNYDGHIAGSLHYASGSFDDKISHLVQN
Consensus  MGRSIFSFFTKKKKMAMARSISYITSTQLLPLHRRPNIAIIDVRDEERNYDGHIAGSLHYASGSFDDKISHLVQN

           76       85        95        105       115       125       135        146
           |--------+---------+---------+---------+---------+---------+---------+|
    Col-0  VKDKDTLVFHCALSQVRGPTCARRLVNYLDEKKEDTGIKNIMILERGFNGWEASGKPVCRCAEVPCKGDCA
    Sij-1  VKDKDTLVFHCALSQVRGPTCARRLVNYLDEKKEDTGIKNIMILERGFNGWEASGKPVCRCAEVPCKGDCA
  Slavi-1  VKDKDTLVFHCALSQVRGPTCARRLVNYLDEKKEDTGIKNIMILERGFNGWEASGKPVCRCAEVPCKGDCA
Consensus  VKDKDTLVFHCALSQVRGPTCARRLVNYLDEKKEDTGIKNIMILERGFNGWEASGKPVCRCAEVPCKGDCA
```

Supplementary Figure 2: Deduced amino acid sequence alignment of AtACR2 in different *Arabidopsis* accessions.
